# Cloning of *chrysanthemum* high-affinity nitrate transporter family (*CmNRT2*) and characterization of *CmNRT2.1*

**DOI:** 10.1038/srep23462

**Published:** 2016-03-23

**Authors:** Chunsun Gu, Aiping Song, Xiaoxue Zhang, Haibin Wang, Ting Li, Yu Chen, Jiafu Jiang, Fadi Chen, Sumei Chen

**Affiliations:** 1 College of Horticulture, Nanjing Agricultural University, Nanjing 210095, China

## Abstract

The family of NITRATE TRANSPORTER 2 (NRT2) proteins belongs to the high affinity transport system (HATS) proteins which acts at low nitrate concentrations. The relevant gene content of the chrysanthemum genome was explored here by isolating the full length sequences of six distinct *CmNRT2* genes. One of these (*CmNRT2.1*) was investigated at the functional level. Its transcription level was inducible by low concentrations of both nitrate and ammonium. A yeast two hybrid assay showed that CmNRT2.1 interacts with CmNAR2, while a BiFC assay demonstrated that the interaction occurs at the plasma membrane. *Arabidopsis thaliana* plants heterologously expressing *CmNRT2.1* displayed an enhanced rate of labeled nitrogen uptake, suggesting that CmNRT2.1 represents a high affinity root nitrate transporter.

Plants have evolved both a high (HATS) and a low (LATS) affinity transport system, which act together to determine nitrate uptake from the soil. The genes involved in HATS and LATS belong to three distinct gene families, namely the nitrate transporter/peptide transporters (*NPFs*), the nitrate transporters (*NRT2s*) and the nitrate assimilation related genes (*NAR2s*)^1^. The *NRT2* family is part of the major facilitator superfamily (MFS). The *Arabidopsis thaliana (A. thaliana)* genome harbors the seven *NRT2* genes, referred to as *AtNRT2.1* through *2.7*^2^,^3^. *NRT2* homologs are also known in other plant species: five have been identified in barley^4^, four in rice^5^ and six in the green alga *Chlamydomonas reinhardtii*^6^. *NRT2* was initially identified as a putative high affinity transport gene in barley^7^ and tobacco^8^ before their presence was revealed in other species including *A. thaliana*^9^,^10^,^11^. On the basis of their transcriptional response to the provision of nitrate, the *AtNRT2*s have been classified into three types: those which are inducible (*AtNRT2.1*, *2.2* and *2.4*), those which are constitutively transcribed (*2.3*, *2.6* and *2.7*), and the repressible gene *2.5*^12^. *AtNRT2.1* encodes a high affinity nitrate transporter which functions at low external nitrate concentrations, acting as either a nitrate sensor or a signal transducer in the root^13^. Although the *AtNRT2.1* product is an important contributor to the inducible component of high affinity nitrate transport^14^, AtNRT2.2 acts as an alternative when AtNRT2.1 is disrupted^15^.

The evidence which implied a functional interaction existed between NRT and NAR proteins was initially acquired in *Chlamydomonas reinhardtii,* but it has also been shown that both the *A. thaliana* mutants *atnrt2.1* and *atnar2.1* lack inducible HATS activity at low levels of nitrate. Direct evidence for the AtNRT2.1/AtNAR2.1 interaction has also been generated via yeast two hybrid, *Xenopus* oocyte and blue native polyacrylamide gel electrophoresis technologies. The loss-of-function of both the rice gene *OsNAR2.1*^16^ and *AtNAR2.1*^17^ leads to a drastic reduction in plant growth.

Chrysanthemum (*Chrysanthemum morifolium*) is a leading ornamental species, particularly in China, where the heavy application of nitrogenous fertilizer is a common practice pursued by growers, despite its known deleterious effect on the environment. So far, only one *CmNRT2* (AB921547 renamed CmNRT2.4 in this study) gene has been isolated; its product interacts with CmNAR2^18^. Here, a description is given of the isolation of a further six *CmNRT2s* and a more detailed analysis of the function of CmNRT2.1 has been given. In particular, its inducibility by both nitrate and ammonium has been characterized, and its interaction with CmNAR2 further detailed. Finally, a demonstration is given that the heterologous expression of *CmNRT2.1* in *A. thaliana* resulted in an increased rate of nitrate uptake into the root.

## Results

### The *CmNRT2s* and their predicted products

**Figure 1:**
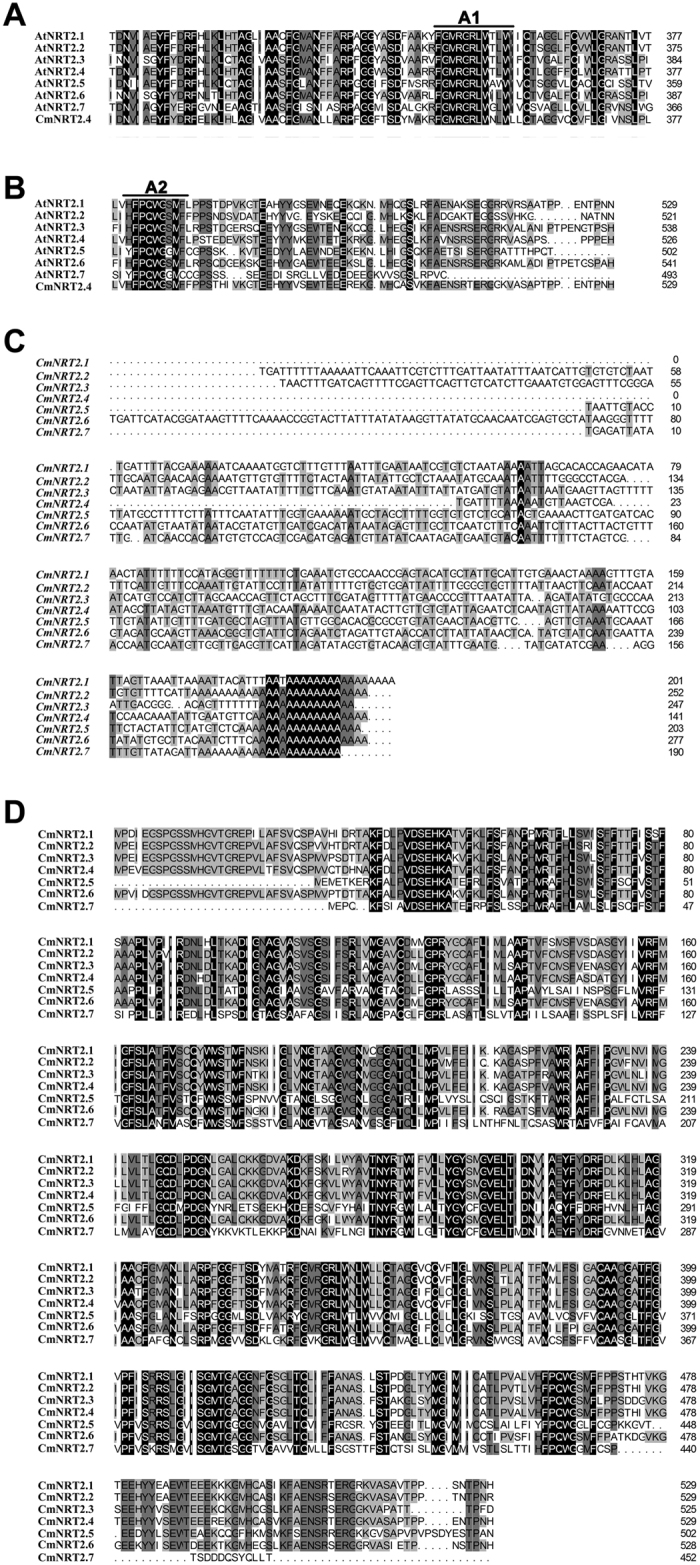
Isolation and peptide-based alignment of *CmNRT2s.* (**A,B**) The two conserved regions identified by aligning the derived amino acid sequences from AtNRT2s and CmNRT2.4; (**C**) an alignment of the *CmNRT2* 3′ UTRs; (**D**) a peptide-based alignment of cloned CmNRT2s.

**Figure 2:**
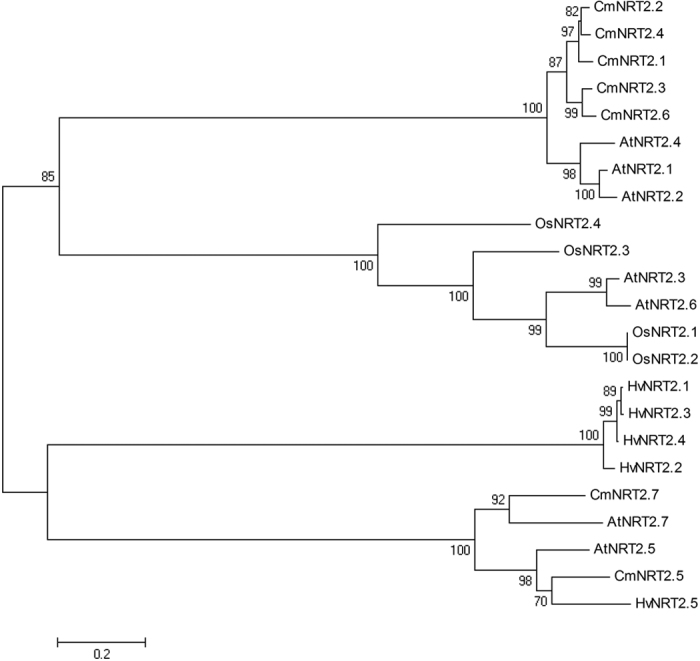
An unrooted phylogenetic tree of the CmNRT2 peptide sequences of chrysanthemum and the barley homologs HvNRT2.1 (U34198), HvNRT2.2 (U34290), HvNRT2.3 (AF091115), HvNRT2.4 (AF091116) and HvNRT2.5 (ABG20828), the rice homologs OsNRT2.1 (P0DKG9), OsNRT2.2 (P0DKH0), OsNRT2.3 (Q94JG1.1) and OsNRT2.4 (A2ZU80.2), the *A. thaliana* homologs AtNRT2.1 (AAC64170), AtNRT2.2 (AAC35884), AtNRT2.3 (BAB10099), AtNRT2.4 (BAB10098), AtNRT2.5 (AAF78499), *AtNRT2.6* (CAB89321) and AtNRT2.7 (CAB87624) and the chrysanthemum homologs CmNRT2.1 (KT203959), CmNRT2.2 (KT203960), CmNRT2.3 (KT203961), CmNRT2.4 (DDBJ accession AB921547), CmNRT2.5 (KT203962), CmNRT2.6 (KT203963) and CmNRT2.7 (KT203964). The sequences were aligned using ClustalW software and the phylogeny constructed using the neighbor-joining method. Five CmNRT2 sequences (2.1, 2.2, 2.3, 2.4, and 2.6) clustered into a single clade supported by a bootstrap value of 100%.

A peptide level sequence comparison between the set of AtNRT2s and the single previously known CmNRT2 (CmNRT2.4, AB921547) revealed the presence of two conserved regions, namely A1 [FGMRGRLW(N/T/A/I/G)(L/W)W] and A2 [(H/Y)FPQWG(S/G)M(F/C)] (Fig. 1A,B). Based on these motifs, the two degenerate primers A1f and A2-2f (Table 1) were designed to amplify the 3′ end of the *CmNRT2*s using 3′ RACE. The outcome of the procedure was the successful identification of seven distinct 3′ untranslated regions (3′ UTRs), including that of *CmNRT2.4* (Fig. 1C), which implied that the chrysanthemum genome housed seven *CmNRT2* genes. The full length cDNAs of *CmNRT2.1* through *2.3* and *2.5* through *2.7* were subsequently obtained by means of 5′ RACE-PCR, following Gu *et al.*^19^. The resulting predicted polypeptide sequences were then aligned (Fig. 1D, Table 2). The greatest extent of sequence identity obtained between CmNRT2.1/2.2 and 2.2/2.4 (94.3%), followed by the 2.1/2.4 pair (93.0%), but only a low level of identity (41.5%) existed between 2.1 and 2.7. The DNA sequences were used to determine the genes’ intron-exon structure^3^ and a schematic representation of the inferred structures is given as Fig. S1. Five of the genes (*2.1*, *2.2*, *2.3*, *2.4* and *2.6*) share two conserved introns, whereas *2.5* harbors two introns sited in a different part of the gene, and *2.7* lacks any introns. A phylogenetic tree based on peptide sequences is shown in Fig. 2; this indicated that five of the gene products (CmNRT2.1, 2.2, 2.3, 2.4 and 2.6) cluster within a single clade with a highly supportive bootstrap value, while 2.5 and 2.7 are outliers. CmNRT2.4 has previously been shown to be a strong candidate as a nitrate uptake protein^18^, so the strong level of peptide similarity and gene structure between it and CmNRT2.1 was suggestive of the latter protein sharing a similar functionality.

### *CmNRT2.1* is inducible by nitrate and ammonium

**Figure 3:**
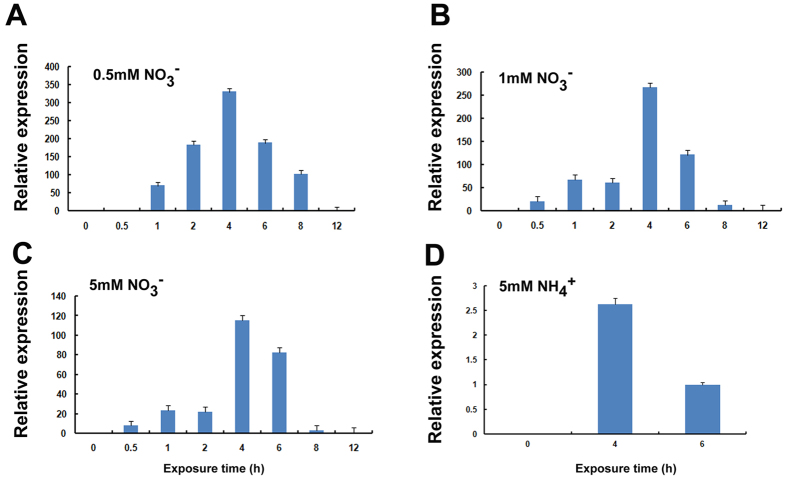
The induction of *CmNRT2.1* transcription in N starved roots exposed to (A) 0.5 mM nitrate, (B) 1 mM nitrate, (C) 5 mM nitrate, (D) 5 mM ammonium, as assayed by qRT-PCR. Error bars in (**A**–**D**) represent the SE (*n = *3).

The topological profiling of *CmNRT2.1* transcription showed that the gene was constitutively transcribed throughout the plant except in the flower, with the root being the site where its transcript was most abundant (Fig. S2). When the plants were provided with various concentrations of either nitrate or ammonium, the abundance of *CmNRT2.1* transcript in the root was enhanced. The nitrate response peaked after 4 h irrespective of the nitrate concentration (Fig. 3A–C), and similarly, the provision of ammonium provoked a transcriptional peak after 4 h (Fig. 3D).

### In *vivo* interaction between CmNRT2.1 and CmNAR2

**Figure 4:**
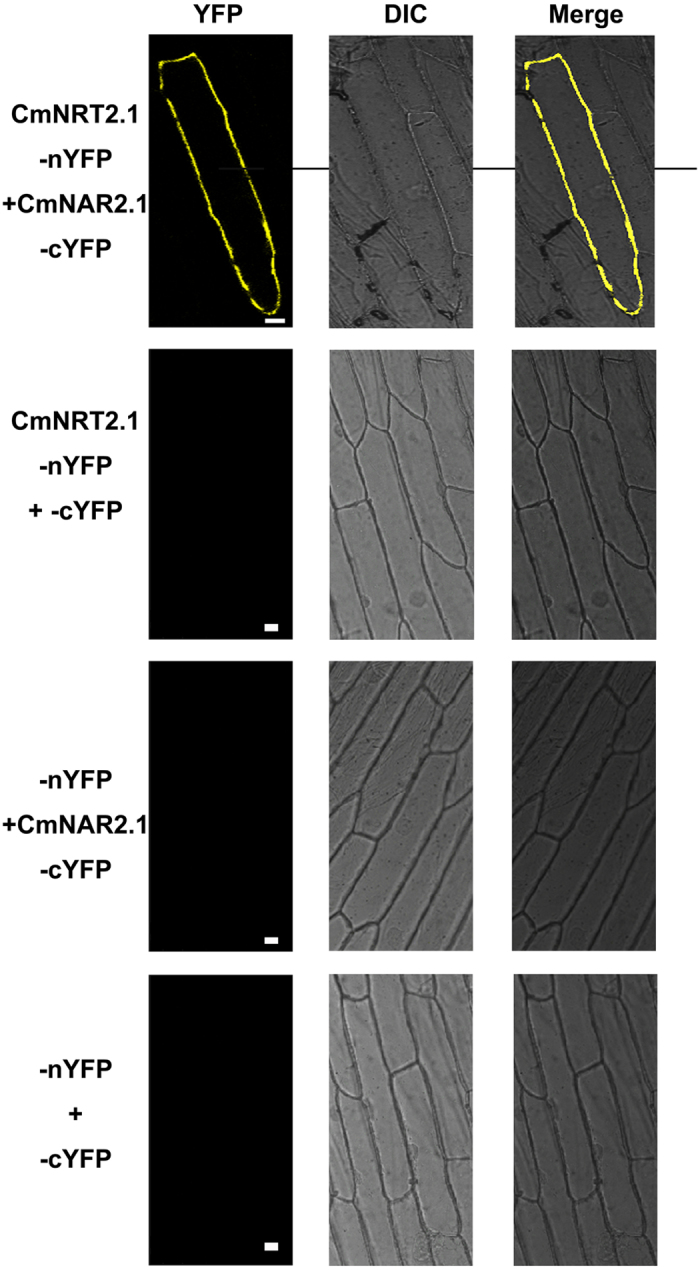
A BiFC analysis of the CmNRT2.1/CmNAR2 interaction in transiently transformed onion epidermal cells. The YFP fluorescence, Differential Interference Contrast (DIC) and merged images are shown for each transgenic combination. Bar: 50 μm.

**Figure 5:**
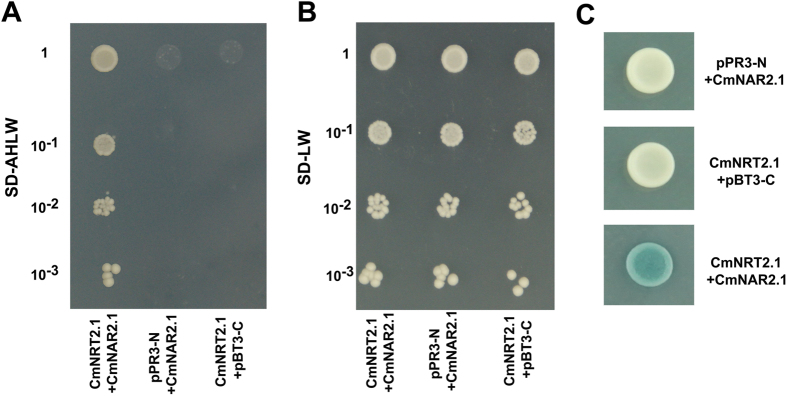
Detection of the CmNRT2.1/CmNAR2 interaction using a membrane pairwise interaction assay. The cells were grown on media lacking (**A**) Ade, His, Leu and Trp, (**B**) Leu and Trp. (**C**) A *β*-galactosidase assay identifies the CmNRT2.1/CmNAR2 interaction. The yeast strain carries both the bait and prey plasmids (pBT3-C and pPR3-N refer to empty *vec*tors). CmNRT2.1, co-transformed with CmNAR2, was used to detect the CmNRT2.1/CmNAR2 interaction. The construct pairs of CmNRT2.1 and CmNAR2 with empty vector, respectively, were used as negative controls.

A BiFC analysis was conducted to characterize the interaction between CmNRT2.1 and CmNAR2, based on the transient expression of split YFP-labelled *CmNRT2*.*1* and *CmNAR2* in onion epidermal cells. The CmNRT2.1 and CmNAR2 fusion proteins were engineered to have complementary N terminal and C terminal EYFP fragments. The epidermal cells expressing *CmNAR2–cEYFP* and *CmNRT2.1–nEYFP* showed strong YFP complementation. In contrast, cells transformed with either *CmNRT2.1-nYFP/cYFP*, *nYFP/CmNAR2.1-cYFP* or *nYFP/cYFP* emitted no fluorescence (Fig. 4). The interpretation of these observations was that CmNRT2.1 can interact with CmNAR2 *in vivo*. As a follow-up experiment, a split-ubiquitin membrane two hybrid system experiment was performed. This confirmed that CmNRT2.1 and CmNAR2 interact, as shown by the ability of the yeast cells to grow on the quadruple dropout medium, while cells carrying either *pPR3-N/pBT3-C-NAR2.1* or *pPR3-N/pBT3-C-NRT2.1* were unable to grow (Fig. 5A). The three combinations used as a positive control all promoted growth on the double-dropout medium (Fig. 5B). When the colonies were re-streaked onto a plate containing X-α-Gal, only cells harboring both *CmNRT2.1* and *CmNAR2* were able to produce a signal (Fig. 5C).

### Transgenic *A. thaliana* lines expressing *CmNRT2.1* showed an enhanced level of nitrate uptake into the root

**Figure 6:**
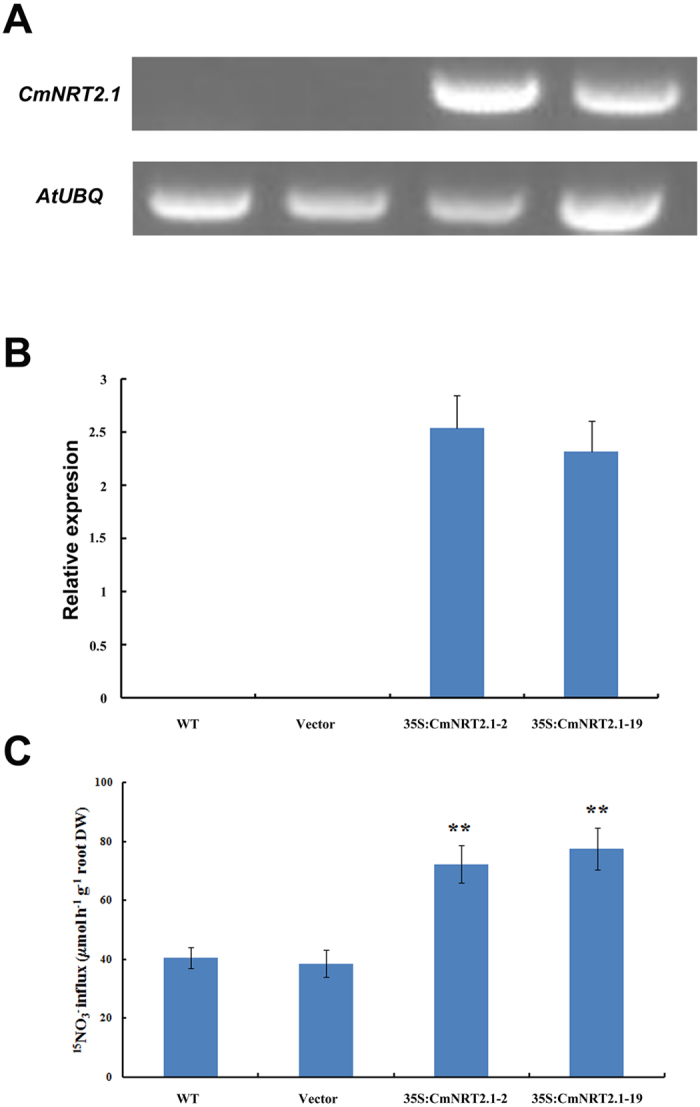
Identification of transgenic *A. thaliana* expressing *CmNRT2.1* and the transgene’s impact on nitrate uptake by the root. (**A**) PCR amplification of genomic DNA identifies the incorporation of the transgene. (**B**) qRT-PCR analysis of mRNA extracted from the transgenic plants. *AtUBQ*: the reference sequence. (**C**) The uptake of labeled ^15^NO_3_ into the root of transgenic and non-transgenic *A. thaliana*. Values represent means ± SE *(n* = 5). Asterisks indicate means which differ significantly (P < 0.01) from the WT value.

*A. thaliana* transgenic plants were generated which constitutively expressed *CmNRT2.1.* An analysis of their genomic DNA (Fig. 6A) and mRNA (Fig. 6B) demonstrated that the transgene was successfully incorporated and transcribed. Two independent transgene homozygous T_3_ selections (RT-2 and -19) were used to study the transgene’s impact on plant growth and nitrate uptake. Both root and shoot fresh weight (FW) of the two lines were significantly higher than those of the wild type (WT) control and vector-transformed lines when the growing medium contained 0.25 mM nitrate. With respect to root FW, there was no significant difference in performance between the transgenics and the controls when the medium contained 10 mM nitrate, but shoot FW was enhanced in RT-2 (Fig. S3). When nitrate uptake was assessed using labeled nitrate, both transgenics out-performed both WT and the empty vector control (Fig. 6C), consistent with the suggestion that CmNRT2.1 provides an improved capacity to take up nitrate.

## Discussion

*NRT2s* have been isolated to date largely using degenerate primers; presently, seven members are known in *A. thaliana*^12^, six in poplar^20^ and four in *Lotus japonicus*^21^. Here, the same approach has been used to identify and isolate six further *CmNRT2s* to add to the one isolated previously^18^. At the peptide level, AtNRT2.1, 2.2 and 2.4 share homology with one another, as do AtNRT2.3 and 2.6. Similarly, a group of four CmNRTs (2.1, 2.4, 2.2 and 2.3) shared closely related sequences (which aligned well with that of AtNRT2.6) (Table 2). It has been shown previously that CmNRT2.4 interacts with CmNAR2 to promote nitrate uptake^18^.

The extensive homology between CmNRT2.1 and 2.4, as well as the nitrate inducibility of *CmNRT2.1* (Table 2) was suggestive of CmNRT2.1 being functionally similar to CmNRT2.4. The level of sequence homology between AtNRT2.1 and CmNRT2.1 was high (>76%). *AtNRT2.1* is inducible by nitrate at a concentration of both 0.5 mM and 10 mM^3^, while the present experiments demonstrated that *CmNRT2.1* was inducible by all three nitrate concentrations provided to the plants. *CmNRT2.1* transcription showed to be induced by the 5 mM ammonium concentrations within 4 h, thereafter was suppressed by 5 mM ammonium. *OsNRT2.1* transcription is similarly suppressed by the presence in the medium of 5 mM ammonium^22^. *CmNRT2.1* transcript was particularly abundant in the root (Fig. S2), a feature which is also characteristic of *AtNRT2.1*^3^. The rice NRT2 homologs *OsNRT2.1, 2.2* and *2.3a* are all induced by the presence of low concentrations of nitrate^22^. In contrast to the transcriptional behavior of *CmNRT2.4*^18^ and the *A. thaliana* genes *AtNRT2.3, 2.6* and *2.7*^12^, *CmNRT2.1* transcript was not detectable when the plants were deprived of either nitrate or ammonium (Fig. 3). Moreover, the increased folds of highest transcription level of *CmNRT2.1* compared to the control is larger than that in *CmNRT2.4* under 4 h nitrate exposure.

The yeast two hybrid and BiFC analyses confirmed that CmNRT2.1 and CmNAR2 interacted with one another *in vivo*, as do a number of the *A. thaliana* NRT2s with AtNAR2.1^23^,^24^, rice NRT2s with OsNAR2.1^16^ and barley NRT2s with HvNAR2.3^10^. Further experiments will be needed to establish whether any of the CmNRT2s other than CmNRT2.1 and 2.4 are able to likewise interact with CmNAR2. The AtNRT2.1/NAR2.1 interaction has been shown to take place in the plasma membrane, forming a 150 kDa complex, thought to act as a high affinity nitrate transporter^24^. The present data were consistent with the CmNRT2.1/NAR2 complex similarly localizing to the plasma membrane (Fig. 4). Transgenic *A. thaliana* plants constitutively expressing *CmNRT2.4* display an enhanced rate of nitrate uptake compared to the WT and the empty vector controls^18^, and the present experiments have shown that CmNRT2.1 activity can also contribute to nitrate uptake (Fig. 6), as expected given the high degree of sequence similarity ( >93%) existing between CmNRT2.1 and 2.4 (Table 2). The conclusion is that *CmNRT2.1* is a nitrate inducible gene, the product of which is a high affinity nitrate transporter. As such, it represents a suitable candidate for the engineering of nitrate uptake efficiency in chrysanthemum.

## Methods

### Plant materials and growth conditions

The experiments were based on the chrysanthemum cultivar ‘Nannongxuefeng’, maintained at the Nanjing Agricultural University Chrysanthemum Germplasm Resource Preserving Centre (Nanjing, China). Phenotypically uniform seedlings at the eight leaf stage were grown in a pH 6.5 medium containing 5 mM NH_4_NO_3_, 2.5 mM K_2_SO_4_, 1.5 mM MgSO_4_.7H_2_O, 1.33 mM NaH_2_PO_4_.2H_2_O, 2.0 mM CaCl_2_, 20 μM H_3_BO_3_, 9 μM MnCl_2_.4H_2_O, 0.77 μM ZnSO_4_·7H_2_O, 0.32 μM CuSO_4_·5H_2_O, 0.39 μM Na_2_MoO_4_.2H_2_O and 20 μM FeNaEDTA, following Gu *et al.*^18^. Nitrification was inhibited by the inclusion of 7 μM dicyandiamide. The solution was refreshed every two days.

### Isolation and sequencing of *CmNRT2* full-length cDNAs

For the gene isolation experiment, seedlings were grown in the solution (as described in the part of plant materials and growing conditions) for four weeks, then starved of nitrogen by removing them to a nitrogen-free version of the same medium for one week. After exposing the seedlings to 5 mM KNO_3_ for 4 h, RNA was extracted from the roots using the RNAiso reagent (TaKaRa, Tokyo, Japan), following the manufacturer’s protocol, then treated with RNase-free DNaseI (TaKaRa, Tokyo, Japan). The concentration and the integrity of the extract were assessed following Gu *et al.*^25^. The first cDNA strand was synthesized using Reverse Transcriptase M-MLV (RNase H^−^) (TaKaRa, Tokyo, Japan), following the manufacturer’s protocol. Two degenerate primers A1f and A2-2f (sequences given in Table 1) were designed based on regions conserved between the *CmNRT2.4* and the *AtNRT2* sequences. The remainder of the cDNA sequence was acquired using RACE-PCR, following Liu *et al.*^26^. Open reading frames (ORFs) were identified from the resulting sequences using ORF finder software (www.ncbi.nlm.nih.gov). The genes’ deduced polypeptide sequences were used in a BLASTp search to identify homologs. An alignment of the seven derived CmNRT2s was performed using DNAman v 5.2.2 software (Lynnon Bio-Soft, Quebec, Canada). A phylogenetic analysis of the CmNRT2s and heterologous NRT2s was finally performed using MEGA 5 software^27^.

### Quantitative real-time PCR (qRT-PCR)

Seedlings were deprived of nitrogen for one week after having been grown in the solution (as described in the part of plant materials and growing conditions) for four weeks. After providing a variable amount of nitrate (0.5, 1.0 or 5.0 mM KNO_3_) or 5 mM NH_4_Cl, the roots were harvested over a time period (0, 0.5, 1, 2, 4, 6, 8 and 12 h for the nitrate experiment and 0, 4 and 6 h for the ammonium experiment), and the RNA extracted as above. RNA was also extracted from various parts of 90-day old plants grown as described in the part of plant materials and growing conditions. Aliquots (1 μg) of the total RNA were reverse transcribed using SuperScript III reverse transcriptase (Invitrogen, Carlsbad, NM, USA) based on oligo(dT) primers. The subsequent qRT-PCRs were formulated with SYBR^®^ Premix *Ex Taq*™ II (TaKaRa). The reactions were initially denatured (95 °C/60 s), then subjected to 40 cycles of 95 °C/15 s, 55 °C/30 s, 72 °C/30 s. A melting curve was obtained by heating the amplicon from 55 °C to 95 °C at 0.5 °C s^−1^. The chosen reference gene was *CmPsaA* (AB548817)^25^. Specific primers of quantitative RT-PCR for *CmNRT2.1* were qCmNRT2.1-F (5′-GTAACACCTCCGAGCAACAC-3′) and qCmNRT2.1-R (5′-TTTCACAATGCAATAGCATG-3′) (Table 1). CmPsaA-F (5′-CCAATAACCACGACCGCTAA-3′) and CmPsaA-R (5′-GGCACAGTCCTCCCAAGTAA-3′) were used to detect the expression level of reference gene of *CmPsaA* (Table 1). The 2^−△△C^_T_ method was used to calculate relative changes in transcript abundance^28^. Each derived relative transcript abundance was based on the mean of three biological replicates.

### Protein-protein interaction assays

The existence of a CmNRT2.1/CmNAR2 interaction was tested using the mating-based split-ubiquitin system (Dualsystems Biotech, Schlieren, Switzerland) and a BiFC assay. The necessary fusion gene p*SAT4A-CmNAR2-cEYFP-N1* has been described previously^18^. For the BiFC analysis, the *CmNRT2.1* ORF was amplified using the primer pair CmNRT2.1-Bi-S/CmNRT2.1-Bi-X (Table 1) and the amplicons of *CmNRT2.1* and *CmNAR2* were used to create the construct p*SAT4A-CmNRT2.1-nEYFP-N1*, p*SAT4A-CmNAR2-cEYFP-N1* and p*SAT4A-CmNRT2.1-nEYFP-N1*. Two constructs were mixed with 1:1 gold particles (Bio-Rad, Hercules, CA) and transformed as described by Gu *et al.*^18^. Confocal laser microscopy was used to monitor the expression of YFP. For the yeast two hybrid analysis, full length *CmNRT2.1* cDNA was first amplified using Phusion^®^ HS DNA polymerase (NEB, Ipswich, MA, US) based on the primer pair pPR3-N-2.1-JS/pPR3-N-2.1-JX (Table 1), and the amplicon introduced into the pPR3-N (TRP1, AmpR) plasmid. The *CmNRT2*.*1* and *CmNAR2* fusions were then co-transformed into yeast strain NMY51 (*MATa his3 trp1 leu2 ade2 LYS2::HIS3 ura3::lacZ ade2::ADE2 GAL4*) using a DS yeast transformation kit (Dualsystems Biotech) following Gu *et al.*^18^. Transformed colonies were selected in SD-LW medium containing 5 mM 3-amino-1,2,4-triazole to enable the selection of positive transformants^18^. Several independent positive transformants were cultured in SD-LW liquid medium at 30 °C overnight. When the OD_546_ of the cultures reached 1.0, the cultures were serially diluted (10×, 100× and 1,000×). A 5 μL aliquot of each dilution was spotted on to both SD-LW and SD-AHLW solid medium and incubated at 30 °C for three days. Positive clones were assayed for β-galactosidase activity.

### Transgene construction and *A. thaliana* transformation

The *CmNRT2.1* ORF was amplified using the primer pair CmNRT2.1-1301-F/CmNRT2.1-1301-R (Table 1), digested with *Bam*HI and *Sac*I and inserted into the pCAMBIA1301-220 *Bam*HI and *Sac*I sites to produce the construct p*1301-220-CmNRT2.1*. Either an empty pCAMBIA1301-220 vector or p1301*-220-CmNRT2.1* was transformed into *Agrobacterium tumefaciens* strain EHA105 using the freeze-thaw method^26^. *A. thaliana* Col-0 was transformed using the floral dip method^18^. T_1_ seedlings were raised on Murashige and Skoog (1962) (MS) medium containing 20 mg/L hygromycin and 25 mg/L ampicillin. Positive transformants were validated by observing GUS expression in the leaf^29^ and also by deploying a PCR assay on their genomic DNA, based on the primer pair CmNRT2.1-F/CmNRT2.1-R (Table 1). qRT-PCR was used to quantify *CmNRT2.1* transcription using the primer pair CmNRT2.1s/CmNRT2.1x (Table 1); the relevant reference gene was *AtUBQ* (NM_116771.5) assayed using the primer pair AtUBQs/AtUBQx (Table 1).

### The performance of transgenic *A. thaliana* expressing *CmNRT2.1*

Seedlings were raised on vertical MS agar plates two weeks, then transferred to a growth culture conditions medium^29^, containing either 0.25 mM nitrate (0.125 mM KNO_3_, 0.0625 mM Ca(NO_3_)_2_) or 10 mM nitrate (5 mM KNO_3_ and 2.5 mM Ca(NO_3_)_2_). The nitrates were replaced by the same molarity of chloride salts to produce a nitrogen deficient medium. Each of three replicated treatments comprised a set of 50 plants. Shoot and root FW was measured after 14 days. The uptake of labeled nitrate (^15^NO_3_) was assayed as described elsewhere^18^. Briefly, the plants were exposed to 0.1 mM CaSO_4_ for 1 min, then to a complete nutrient solution containing 0.2 mM ^15^NO_3_-for 5 min and finally to 0.1 mM CaSO_4_ for 1 min. The root homogenate was dried overnight at 80 °C. The content of labeled nitrate was analyzed using a PDZ Europa ANCA-MS device (Northwich, UK). The recorded measurements represent the mean of three biological repeats.

### Statistical analysis

Statistical analysis was performed by the one-way analysis of variance (ANOVA) using SPSS 11.5 software (SPSS Inc., Champaign, IL), and Duncan’s multiple range test was employed to detect differences between means.

## Additional Information

**How to cite this article**: Gu, C. *et al.* Cloning of *chrysanthemum* high-affinity nitrate transporter family (*CmNRT2*) and characterization of *CmNRT2.1. Sci. Rep.* **6**, 23462; doi: 10.1038/srep23462 (2016).

## Supplementary Material

Supplementary Information

## Figures and Tables

**Table 1:** Sequences of PCR primers used in the study.

Primer	Sequence (5′–3′)
A1f	AGGCGGTTCGGCatgmgnggnmg
A2-2f	CAGAACATGCAGCcccaytgnggra
qCmNRT2.1-F	GTAACACCTCCGAGCAACAC
qCmNRT2.1-R	TTTCACAATGCAATAGCATG
CmPsaA-F	CCAATAACCACGACCGCTAA
CmPsaA-R	GGCACAGTCCTCCCAAGTAA
pPR3-N-2.1-JS	ATTAACAA GGCCATTACGGCCATGCCTGACATTGAAGGT
pPR3-N-2.1-JX	AACTGATT GGCCGAGGCGGCCAACATGGTTAGGGGTGTT
CmNRT2.1-Bi-S	CGGAATTCATGCCTGACATTGAAGGT
CmNRT2.1-Bi-X	CGGGATCCCAACATGGTTAGGGGTGTT
CmNRT2.1-1301-F	GGATCCATGCCTGACATTGAAGGT
CmNRT2.1-1301-R	GAGCTCCAAACATGGTTAGGGGTG
CmNRT2.1-F	ATGCCTGACATTGAAGGT
CmNRT2.1-R	CAAACATGGTTAGGGGTG
CmNRT2.1s	TTTTCTCAATCGGAGCACAAGC
CmNRT2.1x	GCCCTGAACCAAAGTTACCACC
AtUBQs	AGGACAAAGAGGGTATCCCA
AtUBQx	CAGACGCAAGACCAAGTGAA

**Table 2:** Peptide sequence similarity between members of the CmNRT2 family.

	CmNRT2.1						
CmNRT2.1	—	CmNRT2.2					
CmNRT2.2	94.3/97.0	—	CmNRT2.3				
CmNRT2.3	86.8/92.6	88.1/93.6	—	CmNRT2.4			
CmNRT2.4	93.0/96.4	94.3/97.2	88.3/94.0	—	CmNRT2.5		
CmNRT2.5	52.4/72.3	53.8/72.8	53.8/74.1	53.3/72.6	—	CmNRT2.6	
CmNRT2.6	86.0/92.8	86.2/93.0	92.2/95.7	87.0/93.6	53.3/72.8	—	CmNRT2.7
CmNRT2.7	41.5/59.2	42.3/60.0	43.2/60.1	41.7/59.2	53.0/72.4	42.0/59.0	—

Identity/similarity matrix for the CmNRT2 family. Amino Acids Sequence Similarity was identified using the DNAman software package (Version 5.2.2, Lynnon Bio- Soft, Canada).

## References

[b1] Tsay Y. F., Chiu C. C., Tsai C. B., Ho C. H. & Hsu P. K. Nitrate transporters and peptide transporters. FEBS lett. 581, 2290 (2007). 17481610 10.1016/j.febslet.2007.04.047

[b2] Glass A. D. *et al.* Nitrogen transport in plants, with an emphasis on the regulations of fluxes to match plant demand. J. Plant Nutr. Soil Sc. 164, 199–207 (2001).

[b3] Orsel M., Krapp A. & Daniel-Vedele F. Analysis of the NRT2 nitrate transporter family in Arabidopsis. Structure and gene expression. Plant Physiol. 129, 886–896 (2002). 12068127 10.1104/pp.005280 PMC161709

[b4] Vidmar J. J., Zhuo D., Siddiqi M. Y. & Glass A. D. M. Isolation and Characterization of *HvNRT2.3* and *HvNRT2.4*, cDNAs Encoding High-Affinity Nitrate Transporters from Roots of Barley. Plant Physiol. 122, 783–792 (2000). 10712542 10.1104/pp.122.3.783 PMC58914

[b5] Araki R. & Hasegawa H. Expression of rice (Oryza sativa L.) genes involved in high-affinity nitrate transport during the period of nitrate induction. Breeding Sci. 56, 295–302 (2006).

[b6] Fernandez E. & Galvan A. Nitrate assimilation in Chlamydomonas. Eukaryot. cell 7, 555–559 (2008). 18310352 10.1128/EC.00431-07 PMC2292633

[b7] Trueman L. J., Richardson A. & Forde B. G. Molecular cloning of higher plant homologues of the high-affinity nitrate transporters of *Chlamydomonas reinhardtii* and *Aspergillus nidulans*. Gene 175, 223–231 (1996). 8917103 10.1016/0378-1119(96)00154-0

[b8] Quesada A. *et al.* PCR-identification of a Nicotiana plumbaginifolia cDNA homologous to the high-affinity nitrate transporters of the crnA family. Plant Mol. Biol. 34, 265–274 (1997). 9207842 10.1023/a:1005872816881

[b9] Zhuo D., Okamoto M., Vidmar J. J. & Glass A. D. M. Regulation of a putative high-affinity nitrate transporter (*Nrt2;1At*) in roots of *Arabidopsis thaliana*. Plant J. 17, 563–568 (1999). 10205909 10.1046/j.1365-313x.1999.00396.x

[b10] Tong Y., Zhou J. J., Li Z. & Miller A. J. A two-component high-affinity nitrate uptake system in barley. Plant J. 41, 442–450 (2005). 15659102 10.1111/j.1365-313X.2004.02310.x

[b11] Amarasinghe B. R. *et al.* Regulation of GmNRT2 expression and nitrate transport activity in roots of soybean (Glycine max). Planta 206, 44–52 (1998). 9715532 10.1007/s004250050372

[b12] Okamoto M., Vidmar J. J. & Glass A. D. M. Regulation of *NRT1* and *NRT2* gene families of *Arabidopsis thaliana*: responses to nitrate provision. Plant Cell Physiol. 44, 304–317 (2003). 12668777 10.1093/pcp/pcg036

[b13] Little D. Y. *et al.* The putative high-affinity nitrate transporter NRT2.1 represses lateral root initiation in response to nutritional cues. Proc. Natl.Acad. Sci. USA 102, 13693–13698 (2005). 16157886 10.1073/pnas.0504219102 PMC1224627

[b14] Orsel M., Eulenburg K., Krapp A. & Daniel-Vedele F. Disruption of the nitrate transporter genes *AtNRT2.1* and *AtNRT2.2* restricts growth at low external nitrate concentration. Planta 219, 714–721 (2004). 15107992 10.1007/s00425-004-1266-x

[b15] Li W. *et al.* Dissection of the *AtNRT2. 1*: *AtNRT2. 2* inducible high-affinity nitrate transporter gene cluster. Plant Physiol. 143, 425–433 (2007). 17085507 10.1104/pp.106.091223 PMC1761961

[b16] Yan M. *et al.* Rice OsNAR2. 1 interacts with OsNRT2.1, OsNRT2.2 and OsNRT2.3a nitrate transporters to provide uptake over high and low concentration ranges. Plant Cell Environ. 34, 1360–1372 (2011). 21486304 10.1111/j.1365-3040.2011.02335.x

[b17] Okamoto M. *et al.* High-affinity nitrate transport in roots of Arabidopsis depends on expression of the *NAR2*-like gene *AtNRT3.1*. Plant Physiol. 140, 1036–1046 (2006). 16415212 10.1104/pp.105.074385 PMC1400568

[b18] Gu C. *et al.* *Chrysanthemum* CmNAR2 interacts with CmNRT2 in the control of nitrate uptake. Sci. Rep-UK 4, 5833, doi: 10.1038/srep05833 (2014). PMC5376060 25060485

[b19] Gu C. *et al.* Overexpression of *Iris. lactea* var. *chinensis* metallothionein llMT2a enhances cadmium tolerance in *Arabidopsis thaliana*. Ecotox. Environ. Safe. 105, 22–28 (2014). 10.1016/j.ecoenv.2014.04.002 24780229

[b20] Bai H., Euring D., Volmer K., Janz D. & Polle A. The Nitrate Transporter (NRT) Gene Family in Poplar. PloS one 8, doi: e72126 10.1371/journal.pone.0072126 (2013). PMC3747271 23977227

[b21] Criscuolo G., Valkov V. T., Parlati A., ALVES L. & Chiurazzi M. Molecular characterization of the *Lotus japonicus NRT1 (PTR)* and *NRT2* families. Plant Cell Environ. 35, 1567–1581 (2012). 22458810 10.1111/j.1365-3040.2012.02510.x

[b22] Feng H. *et al.* Spatial expression and regulation of rice high-affinity nitrate transporters by nitrogen and carbon status. J. Exp. Bot. 62, 2319–2332 (2011). 21220781 10.1093/jxb/erq403

[b23] Kotur Z. *et al.* Nitrate transport capacity of the Arabidopsis thaliana NRT2 family members and their interactions with AtNAR2. 1. New Phytol. 194, 724–731 (2012). 22432443 10.1111/j.1469-8137.2012.04094.x

[b24] Yong Z., Kotur Z. & Glass A. D. M. Characterization of an intact two-component high-affinity nitrate transporter from Arabidopsis roots. Plant J. 63, 739–748 (2010). 20561257 10.1111/j.1365-313X.2010.04278.x

[b25] Gu C. *et al.* Reference gene selection for quantitative real-time PCR in Chrysanthemum subjected to biotic and abiotic stress. Mol. Biotechnol. 49, 192–197 (2011). 21416201 10.1007/s12033-011-9394-6

[b26] Liu Z. *et al.* Heterologous expression of a *Nelumbo nucifera* phytochelatin synthase gene enhances cadmium tolerance in *Arabidopsis thaliana*. Appl. Biochem. Biotech. 166, 722–734 (2012). 10.1007/s12010-011-9461-2 22161260

[b27] Tamura K. *et al.* MEGA5: Molecular Evolutionary Genetics Analysis Using Maximum Likelihood, Evolutionary Distance, and Maximum Parsimony Methods. Mol. Biol. Evol. 28, 2731–2739 (2011). 21546353 10.1093/molbev/msr121 PMC3203626

[b28] Livak K. J. & Schmittgen T. D. Analysis of relative gene expression data using real-time quantitative PCR and the 2−ΔΔCT method. Methods 25, 402–408 (2001). 11846609 10.1006/meth.2001.1262

[b29] Remans T. *et al.* A central role for the nitrate transporter NRT2.1 in the integrated morphological and physiological responses of the root system to nitrogen limitation in Arabidopsis. Plant Physiol. 140, 909–921 (2006). 16415211 10.1104/pp.105.075721 PMC1400583

