# Supplementary material for: Cloning of chrysanthemum high-affinity nitrate transporter family (CmNRT2) and characterization of CmNRT2.1
Source: Sci Rep. 2016 Mar 23;6:23462. doi: 10.1038/srep23462 (PMC4804277; doi:10.1038/srep23462)

# Title:Cloning of *chrysanthemum*high-affinity nitrate transporter family (*CmNRT2*) and characterization of *CmNRT2.1*

**Authors:** Chunsun Gu, Aiping Song, Xiaoxue Zhang, Haibin Wang, Ting Li, Yu Chen, Jiafu Jiang, Fadi Chen,Sumei Chen^§^

**Fig. S1** Gene structure of the *CmNRT2* family.Box and black lines represent exons and introns respectively.


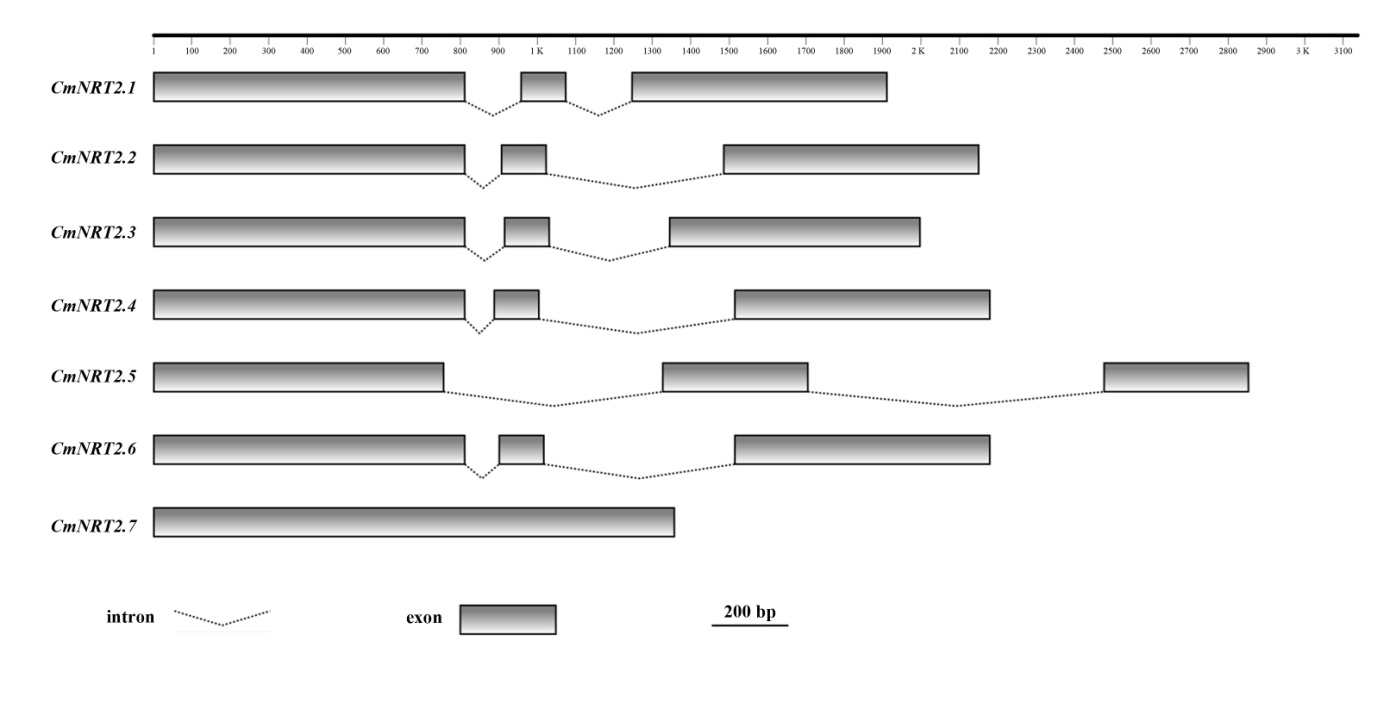


**Fig. S2** The induced transcription of*CmNRT2.1* in root, leaf, stem and flower. ND represents no detectable expression. Error bars represents SD of three biological repeats.


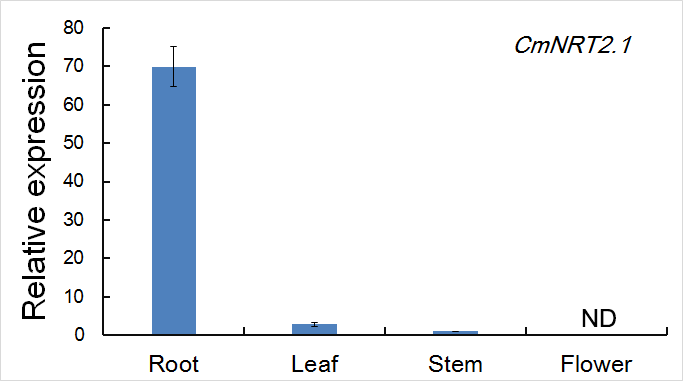


**Fig. S3** Fresh weight comparison of *CmNRT2.1* overexpressing *A. thaliana* and non-transformed ones. The experiment was replicated three times, with each replicate consisting of 50 seedlings. Duncan’s multiple range test (with P set at 0.05) was employed to detect differences between means.


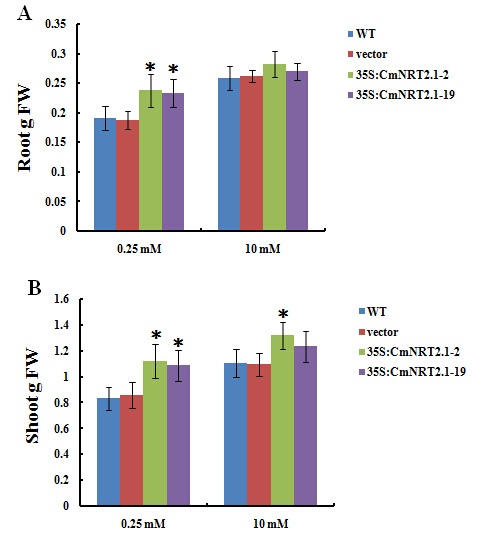

Supplement: Supplementary Information [file srep23462-s1.doc]
